# Adolescents’ psychological health during the economic recession: does public spending buffer health inequalities among young people?

**DOI:** 10.1186/s12889-016-3551-6

**Published:** 2016-08-24

**Authors:** Katharina Rathmann, Timo-Kolja Pförtner, Ana M. Osorio, Klaus Hurrelmann, Frank J. Elgar, Lucia Bosakova, Matthias Richter

**Affiliations:** 1 Institute of Medical Sociology, Medical Faculty, Martin Luther University Halle-Wittenberg, Magdeburger Str. 8, 06112 Halle, Germany; 2 Institute of Medical Sociology, Health Services Research, and Rehabilitation Science, Faculty of Human Sciences and the Faculty of Medicine, University of Cologne, Cologne, Germany; 3 Pontificia Universidad Javeriana Cali, Cali, Colombia; 4 Hertie School of Governance Berlin, Berlin, Germany; 5 Institute for Health and Social Policy, McGill University, Montreal, QC Canada; 6 Department of Health Psychology, Medical Faculty, P. J. Safarik University in Kosice, Kosice, Slovak Republic; 7 Department of Quantitative Methods, Faculty of Business Economy in Kosice, University of Economics in Bratislava, Bratislava, Slovak Republic; 8 Olomouc University Social Health Institute (OUSHI), Palacky University in Olomouc, Olomouc, Czech Republic

**Keywords:** Health inequalities, Recession, Social spending, Adolescence, Multilevel analysis

## Abstract

**Background:**

Many OECD countries have replied to economic recessions with an adaption in public spending on social benefits for families and young people in need. So far, no study has examined the impact of public social spending during the recent economic recession on health, and social inequalities in health among young people. This study investigates whether an increase in public spending relates to a lower prevalence in health complaints and buffers health inequalities among adolescents.

**Methods:**

Data were obtained from the 2009/2010 “Health Behaviour in School-aged Children (HBSC)” study comprising 11 – 15-year-old adolescents from 27 European countries (*N* = 144,754). Socioeconomic position was measured by the Family Affluence Scale (FAS). Logistic multilevel models were conducted for the association between the absolute rate of public spending on family benefits per capita in 2010 and the relative change rate in family benefits (2006–2010) in relation to adolescent psychological health complaints in 2009/2010.

**Results:**

The absolute rate of public spending on family benefits in 2010 did not show a significant association with adolescents’ psychological health complaints. Relative change rates of public spending on family benefits (2006–2010) were related to better health. Greater socioeconomic inequalities in psychological health complaints were found for countries with higher change rates in public spending on family benefits (2006–2010).

**Conclusions:**

The results partially support our hypothesis and highlight that policy initiatives in terms of an increase in family benefits might partially benefit adolescent health, but tend to widen social inequalities in adolescent health during the recent recession.

**Electronic supplementary material:**

The online version of this article (doi:10.1186/s12889-016-3551-6) contains supplementary material, which is available to authorized users.

## Background

The recent economic recession has had a detrimental impact on health and supposedly on social disparities in health in many countries [1, 2]. For adult populations, prior evidence revealed that recessions can have detrimental effects on many health outcomes [3–10]. Other studies showed positive health outcomes during times of economic crises, particularly for wealthy countries [11, 12]. As a political response to the recent economic recession, many countries in Europe have reacted by leveling up or cutting expenditures for social welfare programs such as family benefits. The aim of these programs is to guarantee social security for families and (young) people in need by maintaining the standard of living for people at risk and attempting to hold the extent of social inequalities within societies at a moderate level [5, 9, 10].

Single-country studies have shown that social welfare programs can mitigate some negative effects of the recession on health, such as suicides and other mental and physical health problems [2, 4, 11, 13]. Other studies indicated that investment in measures to support the well-being of parents and their children can protect mental health, with long-term economic gains outweighing short-term costs [10, 14]. Thus, it is likely that social benefits play a protective role for health during economic changes. With regard to health inequalities, some national studies found that health inequalities have not necessarily widened during a recession in countries with good formal social protection [4, 15].

However, little is known about the impact of the current economic recession on young people’s health and on health inequalities from a cross-national perspective as previous studies focused mostly on adults in single countries or on previous recessions [16, 17]. Even less is known about the role of recession-related outcomes, such as unemployment rates or social spending, in relation to young people’s health inequalities. The aims of this study are therefore 1) to investigate the impact of public spending on family benefits (in 2010) and the change rate in public spending on family benefits (2006–2010) on adolescent subjective health, and 2) to examine whether the absolute level of family benefits (2010) and change rate in family benefits (2006–2010) mitigated the extent of social inequalities in adolescent health. Further, this study follows two assumptions: a) countries which have higher public spending on family benefits in 2010 (during the recession) have better adolescent health and smaller inequalities in health, and b) increased public spending on family benefits during the current recession (2006–2010) ameliorate adolescents’ health and reduced social inequalities in health.

## Methods

### Data

Data were obtained from the Health Behaviour in School-aged Children (HBSC) studies in 2005/2006 and 2009/2010. The HBSC-study is a cross-national survey conducted in collaboration with the World Health Organization. The objective of the study is to investigate health, health behaviours and their social determinants among 11 – 13- and 15-year old adolescents [15, 18]. Research groups in 41 countries in the Europe, North America and Israel took part in the 2005/2006 and 2009/2010 survey, using a standardized questionnaire and adhering to an internationally agreed protocol [15]. Data were collected by means of standardized questionnaires, administered in school classrooms according to standardized instructions. The response rate at the school level was above 80 % in the majority of the countries. Ethical approval was obtained for each national survey according to the national guidance and regulation at the time of data collection. In some of the countries participating in the HBSC study ethical approval was not necessary or applicable at the time. In other countries that required approval this was obtained by different Institutional Review Boards (see Additional file 1). Unfortunately, HBSC survey data are not available as scientific use files. The present analysis was based on 27 out of 41 countries (for HBSC 2005/06: *N* = 144,341 and for HBSC 2009/2010: *N* = 144,754). England, Wales and Scotland form one country. Data from French and Flemish regions of Belgium were also combined. Several countries were excluded from the analysis due to missing data on public spending on family benefits in 2005/2006 and 2009/2010 (Croatia, Greenland, Malta, Macedonia, Turkey and Ukraine). Further, we excluded Canada, Israel and USA as they do not belong to the European Union and were, hence, not represented in the Eurostat Databank. Individual data from adolescents in Bulgaria were not available for the HBSC survey in 2009/2010. Missing values for the individual-level variables were excluded from the analyses. Table 1 shows the sample size for each country and all indicators used.

**Table 1:** Description of data and sample (HBSC 2005/2006 and 2009/2010, EUROSTAT, OECD SOCX)

Sample			Macro-level indicators				Outcome			Individual-level indicators			
			Public spending on family benefits per capita		National wealth	Youth unemployment	Two or more psychological health complaints (%)			family affluence mean (range: 0 = low – 9 = high)		Gender: girls	mean age
Country (*N* = 27)	*N* 2005/2006	*N* 2009/2010	2009/2010	change rate between 2005/2006 and 2009/2010 (%)	GDP per capita, 2009/2010	2009/2010 (%)	2005/2006	2009/2010	change rate between 2005/2006 and 2009/2010	2005/2006	2009/2010	(2009/2010) in %	2009/2010 in years
Austria	4,395	4,693	926.0	9.9	35,056	9.4	10.2	12.6	24.0	5.6	6.0	51,7	13,5
Belgium	7,860	7,155	615.4	12.3	32,648	22.2	19.8	18.3	-7.7	5.7	6.2	50,9	13,4
Czech Rep.	4,610	4,199	281.8	8.7	23,369	17.5	26.2	29.7	13.3	4.7	5.5	51,7	13,5
Denmark	5,288	3,852	1258.0	23.0	32,176	12.9	15.0	14.7	-1.8	6.1	6.6	53,0	13,5
Estonia	4,288	4,087	346.3	56.4	16,403	30.2	22.6	21.5	-5.2	4.7	5.8	52,6	13,8
Finland	5,003	6,390	884.6	18.7	30,881	21.5	18.8	17.6	-6.7	5.5	6.1	52,5	13,6
France	6,816	5,773	710.3	8.3	29,457	23.5	25.5	23.7	-7.0	5.8	6.4	50,8	13,4
Germany	6,944	4,775	910.1	13.5	32,850	10.6	13.2	11.9	-10.5	5.7	6.2	51,7	13,4
Greece	3,601	4,668	396.8	27.0	24,923	29.4	34.9	31.9	-8.6	4.9	5.5	52,4	13,7
Hungary	3,379	4,678	491.0	19.8	16,821	26.6	24.5	21.2	-13.5	4.8	5.1	53,2	13,6
Iceland	9,007	10,573	872.7	0.3	33,393	16.1	21.5	19.8	-7.9	7.0	7.1	50,2	13,5
Ireland	4,405	4,005	1014.2	27.8	35,762	25.8	17.3	20.5	18.6	4.7	5.7	47,9	13,7
Italy	3,802	4,626	342.1	24.9	26,894	26.6	34.9	33.0	-5.2	5.1	5.7	50,4	13,4
Latvia	4,040	3,958	147.9	37.9	17,261	34.8	26.9	23.3	-13.3	4.6	5.1	53,0	13,6
Lithuania	5,394	5,103	363.2	137.6	18,845	32.7	26.7	25.6	-4.3	4.4	5.2	49,5	13,7
Luxembourg	4,001	3,885	2295.0	17.2	67,264	16.2	23.0	21.2	-7.8	6.2	6.6	50,2	13,6
Netherlands	4,033	4,259	401.1	-4.4	36,713	8.2	13.3	14.5	9.4	6.0	6.6	51,5	13,5
Norway	4,381	4,174	1263.5	19.3	46,964	9.2	17.5	19.3	10.3	6.8	7.2	50,1	13,4
Poland	5,374	4,063	122.4	21.5	16,954	22.2	27.5	27.5	0.0	4.7	5.3	52,1	13,7
Portugal	3,734	3,852	260.4	24.2	21,578	26.7	15.1	16.6	9.7	5.0	6.0	54,0	13,7
Romania	4,322	4,792	199.3	30.9	15,921	21.5	33.2	30.1	-9.5	3.9	4.3	51,8	13,3
Slovakia	3,533	4,685	315.8	28.2	19,762	30.8	31.5	27.3	-13.3	4.0	5.0	52,9	13,7
Slovenia	4,916	5,261	430.6	13.7	24,897	14.2	14.2	11.1	-22.2	5.7	6.3	49,8	13,6
Spain	8,563	4,817	351.6	24.6	26,976	39.6	22.2	22.9	2.8	5.5	6.1	51,4	13,8
Sweden	4,196	6,195	912.6	13.2	33,211	24.9	22.0	20.6	-6.3	6.2	6.4	51,2	13,5
Switzerland	4,394	6,371	423.4	23.5	38,637	6.1	20.1	19.4	-3.2	5.8	6.3	50,1	13,6
UK	14,062	13,865	481.8	10.5	32,625	19.4	20.0	20.1	0.1	5.8	6.1	52,8	13,7
Total	144,341	144,754	625.7	22.0	29,646	21.0	22.1	21.3	-3.7	5.5	6.0	51,5	13,6

Note: The indicator on public spending on family benefits per capita is measured as constant prices, constant PPP, in Euros

### Indicators

#### Mental health status

The indicator used to identify the health status of adolescents in the analysis was weekly psychological health complaints [19]. Health complaints were measured using the HBSC symptom check list [20]. Students were asked to indicate how often they had experienced the following psychological symptoms in the last six months: irritable or bad tempered, feeling nervous, difficulties in getting to sleep and feeling dizzy. The response options were “almost daily“, “several times per week“, “almost every week“, “about once per month“, “rarely or never.” A sum index, indicating the number of at least weekly psychological health complaints, was calculated from the four items (range: 0–4 health complaints several times per week). Lastly, this index was dichotomised (1 = 2+ psychological health complaints at least weekly vs. 0 = <2 complaints).

#### Socioeconomic position of adolescents’ family of origin

The socioeconomic position of adolescents was measured by the Family Affluence Scale (FAS) which was based on four items [18]. “Does your family own a car, van, or truck?” (0 = *no*, 1 = *yes, one*, 2 = *yes, two or more*), “Do you have your own bedroom for yourself?” (0 = *no*, 1 = *yes*), “During the past 12 months, how many times did you travel away on holiday with your family?” (0 = *not at all*, 1 = *once*, 2 = *twice*, 3 = *more than twice*), and “How many computers does your family own?” (0 = *none*, 1 = *one*, 2 = *two*, 3 = *more than two*). The scores of the answers were summed (range 0–9), and were grouped into high (7–9), medium (4–6), and low (0–3) FAS tertiles [18]. The FAS has been validated across European and North American countries and can be used as “an indicator of child material affluence” [18].

#### Social policy response to the economic recession

We used public spending on family benefits per capita (in constant prices and constant purchasing power parity, PPP) as a measure of social policy response to the economic recession. The country data was available for 28 European Union member states via Eurostat databank (https://eurostat.ec.europa.eu). We included two measures of public spending on family benefits: the absolute rate of family benefits in 2009/2010 (during the recession) in order to assess the current level of national public spending on family benefits, respectively, as well as the relative change rate in family benefits (2005/2006–2009/2010) in order to indicate the response level to the recent economic recession. All country-level variables were centred on the grand mean across all 27 countries.

#### Control variables

As control variables, we considered national wealth (measured by the Gross Domestic Product in 2010, GDP) in order to control for differences in absolute wealth among countries, using country’s GDP per capita in 2009/2010 (in constant prices and constant purchasing power parity (PPP), in Euros) (http://data.worldbank.org). We further controlled for youth unemployment rate in 2009/2010 as unemployment rates have often been used in previous health studies as an indicator of recessions [4, 21]. EUROSTAT provides data on unemployment among young people between 15 and 24 years which refer to the number of persons who are unemployed as a percentage of the total number of employed and unemployed persons [22]. At the individual level we used students’ age and gender as control variables.

### Statistical analyses

The study utilizes multilevel analysis that allows the modelling of hierarchical or nested data structures. The level 1-units in the sample are individual students; the level 2-units are the 27 countries. Multilevel analysis is based on the assumption that both the regression constant (intercept) and the regression coefficients of the individual predictors (slope) may vary for individuals between countries, and may be explained by country-level characteristics. By using psychological health complaints as the outcome, we examined – by conducting random intercept models [23] – the extent to which the intercept of the outcome differed among the 27 countries. We further simultaneously included country-level indicators of public spending on family benefits that might explain this variation in psychological health complaints as well as in the extent of social inequalities in complaints across countries.

The individual- and country-level determinants were included in the models using a stepwise approach. First, an empty model (model 1) tested the Intraclass Correlation Coefficient (ICC), which represents the proportion of variance on latent country effects by indicating the variance in the outcome attributed to country differences. Model 2 considered individual-level variables. Model 3 included both indicators of family benefits, simultaneously, controlled for youth unemployment rate and national wealth in 2009/2010, respectively. We included cross-level interaction terms between gender and both measures of family benefits (model 4) in order to examine whether the impact of public spending on family benefits and the change rate in family benefits on psychological health complaints differs among boys and girls. Finally, model 5 considered all country-level indicators as well as cross-level interaction terms between family affluence and both country-level indicators of family benefits. The statistical analyses were conducted using the software Stata 12.1 (StataCorp LP, College Station, TX).

## Results

### Descriptive results

The pairwise correlations among all country-level indicators of family benefits (Table 2), youth unemployment rate and national wealth, showing that family benefits in 2009/2010 is highly and positively correlated with national wealth (GDP per capita) in 2009/2010 (r = 0.87, *p* <0.001).

**Table 2:** Pairwise correlations between country-level indicators (N_Country_ = 27)

	Family benefits in 2009/2010	Change rate in family benefits (2005/2006–2009/2010)	National wealth (GDP per capita) in 2009/2010	Youth unemployment rate in 2009/2010
Family benefits in 2009/2010	1			
Change rate in family benefits (2005/2006–2009/2010)	-0.21	1		
National wealth (GDP per capita) in 2009/2010	0.87*	-0.35	1	
Youth unemployment rate in 2009/2010	-0.41	0.48	-0.56	1

Note: * significant at *p* < 0.001

Around 20 % of all adolescents report to have two or more psychological health complaints in 2009/2010 (Table 1). The prevalence rates of complaints in 2009/2010 (Table 1) were lowest in Germany (11.9 %) and Slovenia (11.1 %) and highest in Greece (31.9 %) and Italy (33.0 %). A slight decrease in complaints was found in all European countries (2005/2006–2009/2010). Adolescents in Slovenia (-22.2 %) had the highest decrease in the prevalence of complaints, whereas Austria (+24.0 %) and Ireland (+18.6 %) revealed the highest increase (2005/2006–2009/2010).

The lowest levels of public spending on family benefits per capita in 2009/2010 (Table 1) was found in Poland, Romania, Latvia and Portugal, while Luxembourg, Norway and Denmark revealed the highest levels. Many OECD countries responded to the current economic recession by increasing public spending on family benefits in order to guarantee social security for families and young people in need [9]. This trend can be observed by the positive relative change rates in public spending on family benefits per capita (2005/2006–2009/2010), indicating that family benefits per capita increased in the majority of countries, with the highest relative increase in Lithuania (137.6 %) and Estonia (+56.4 %) and a slight decrease in the Netherlands (-4.4 %).

**Fig. 1:**
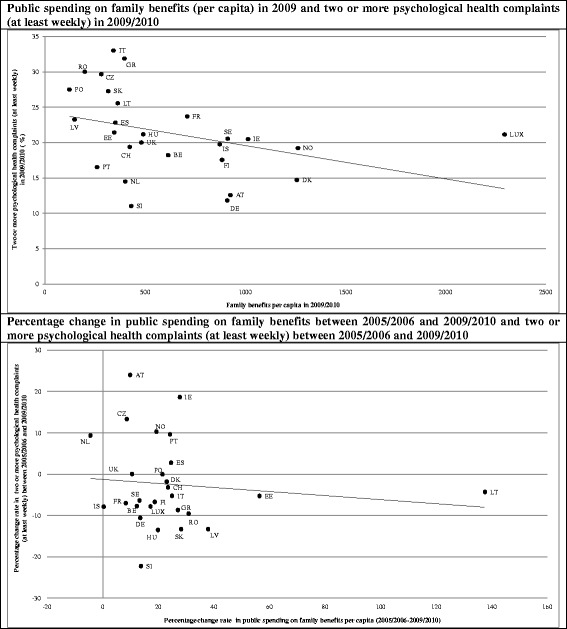
Associations between average level (2009/2010) and change rate (%) of public spending on family benefits* (2005/2006-2009/2010) in relation to the magnitude and change rate in two or more psychological health complaints (in %) across 27 European countries (HBSC 2005/2006 and 2009/2010, EUROSTAT, OECD SOCX). Note: * The indicator of public expenditures on family benefits per capita is measured as constant prices and constant purchasing power parity (in Euros). Labels for countries: Austria (AT), Belgium (BE), Czech Republic (CZ), Denmark (DK), Estonia (EE), Finland (FI), France (FR), Germany (DE), Greece (GR), Hungary (HU), Iceland (IS), Ireland (IE), Italy (IT), Latvia (LV), Lithuania (LT), Luxembourg (LUX), Netherlands (NL), Norway (NO), Poland (PO), Portugal (PT), Romania (RO), Slovakia (SK), Slovenia (SI), Spain (ES), Sweden (SE), Switzerland (CH) and the United Kingdom (UK)

Figure 1 shows the associations between the absolute level of public spending on family benefits and complaints in 2009/2010 as well as the correlation between the increase in public spending on family benefits (2005/2006–2009/2010) and the change rate in complaints (2005/2006–2009/2010), respectively. According to the average level of public spending on family benefits in 2009/2010, weak correlations could be found with complaints, indicating that adolescents living in countries with higher degrees of public spending on family benefits in 2009/2010 tend to display lower prevalence rates in complaints (Pearson R^2^ ~ 0.05). A similar tendency was found for the association between the relative increase in family benefits (2005/2006–2009/2010) and the relative change rate in complaints (2005/2006–2009/2010) (Pearson R^2^ ~ 0.14).

### Multilevel results

Table 3 presents the logistic multilevel regression results for weekly psychological health complaints in 2009/2010 across 27 European countries. Model 1 indicates the variance in psychological health complaints among countries (ICC = 3.75 %). The likelihoods of reporting two or more weekly psychological health complaints increased with age and were higher for medium- and low-affluent adolescents compared to the youngest age group (11 years) and students with high affluence (model 2), respectively. In general, likelihoods of having reported health complaints were higher for girls compared to their male counterparts.

**Table 3:** Associations between family benefits^a^ (2009/2010), percentage change in family benefits^a^ (2005/2006–2009/2010) and psychological health complaints (HBSC 2009/2010)

	Empty model (M1)	Individual variables (M2)	Macro-level variables (M3)	Model with cross-level interactions with gender (M4)	Model with cross-level interactions with FAS (M5)
	OR (95 % CI)	OR (95 % CI)	OR (95 % CI)	OR (95 % CI)	OR (95 % CI)
Individual variables					
Sex (Ref.: *boys*)		1	1	-	1
Girls		1.708***	1.708***	-	1.708***
		(1.66-1.75)	(1.66-1.75)	-	(1.66-1.75)
Age (Ref.: *11 years*)		1.000	1.000	1.000	1.000
13 years		1.289***	1.289***	1.289***	1.289***
		(1.25-1.33)	(1.25-1.33)	(1.25-1.33)	(1.25-1.33)
15 years		1.486***	1.486***	1.486***	1.486***
		(1.44-1.54)	(1.44-1.54)	(1.44-1.54)	(1.44-1.54)
Family affluence (Ref.: *high*)		1.000	1.000	1.000	1.000
Medium		1.129***	1.129***	1.130***	1.129***
		(1.10-1.17)	(1.10-1.17)	(1.096-1.17)	(1.10-1.17)
Low		1.386***	1.386***	1.387***	1.386***
		(1.34-1.43)	(1.34-1.43)	(1.34-1.43)	(1.34-1.43)
Macro-level variables					
National wealth in 2009/2010 (GDP pc)			1.000 (0.99-1.00)	1.000 (0.99-1.00)	1.000 (0.99-1.00)
Youth unemployment rate (2009/2010)			1.024*** (1.01-1.04)	1.024*** (1.01-1.04)	1.024*** (1.01-1.04)
Family benefits in 2009/2010			1.000	0.999	0.999
			(0.99-1.00)	(0.99-1.00)	(0.99-1.00)
relative change in family benefits^a^ (2005/2006-2009/2010)			0.999* (0.98-0.99)	1.001 (0.99-1.01)	0.999* (0.98-0.99)
Cross-level interactions					
Family benefits in 2009/2010 x girls (Ref.: boys)			-	1.001*** (1.00-1.01)	-
relative change in family benefits^a^ (2005/2006-2009/2010) x girls (Ref.: boys)			-	1.001* (1.00-1.02)	-
Family benefits in 2009/2010 x high FAS (Ref.)					1.000
x medium FAS				-	1.000
					(0.99-1.01)
x low FAS				-	1.000
					(0.99-1.01)
relative change in family benefits^a^ (2005/2006-2009/2010) x high FAS (Ref.)					1.000
x medium FAS				-	1.002**
					(1.00-1.01)
x low FAS				-	1.004***
					(1.00-1.01)
Constant	0.261*** (0.23-0.30)	0.136*** (0.12-0.16)	0.136*** (0.12-0.15)	0.136*** (0.12-0.15)	0.136*** (0.12-0.15)
ICC (country-level)	0.0375 = 3.75 %	0.0386 = 3.85 %	0.0247 = 2.47 %	0.0246 = 2,46 %	0.0244 = 2.44 %
N (Individuals)	144,754	144,754	144,754	144,754	144,754
N (Countries)	27	27	27	27	27

Note: * *p* < 0.05, ** *p* < 0.01, *** *p* < 0.001. Country-level indicators are centered on the Grand-Mean. ^**a**^ Public expenditures on family benefits were measured per capita in constant prices and constant purchasing power parity (in Euros) (Source: EUROSTAT)

These associations were not attenuated when country-level indicators have been included (models 3 and 4). Model 3 considers the family benefits rate in 2009/2010 (during the recession) and the relative change rate in family benefits (2005/2006–2009/2010), besides the country-level control variables of youth unemployment in 2009/2010 and national wealth (GDP per capita, 2009/2010).

**Fig. 2:**
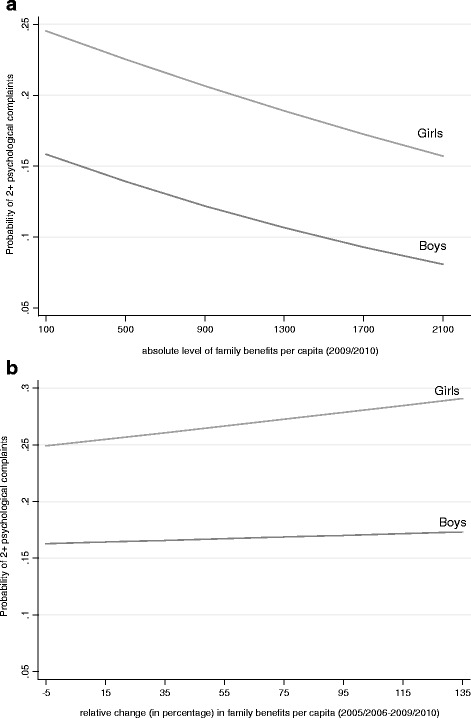
**a** Predicted probabilities of two or more (at least weekly) psychological complaints (*N* = 144,754), according to the absolute level of public spending on family benefits* (2005/2006-2009/2010) across 27 countries, stratified by gender, HBSC 2009/2010. Note: *The indicator of public expenditures on family benefits per capita is measured as constant prices and constant purchasing power parity (in Euros). **b** Predicted probabilities of two or more (at least weekly) psychological complaints (*N* = 144,754), according to the relative percentage change in family benefits* (2005/2006-2009/2010) across 27 countries, stratified by gender, HBSC 2009/2010. Note: *The indicator of public expenditures on family benefits per capita is measured as constant prices and constant purchasing power parity (in Euros)

The results show that the absolute level of family benefits in 2009/2010 was not significantly associated with the likelihood of health complaints, whereas adolescents in countries with higher increases in family benefits (2005/2006–2009/2010) showed lower risks of reporting two or more health complaints (OR: 0.999, 95 % CI: 0.98–0.99). Further, higher youth unemployment rates were significantly related to a higher risk of reporting two or more complaints (OR: 1.024, 95 % CI: 1.01–1.04), whereas national wealth had no association with the outcome. In order to examine whether the impact of public spending on family benefits and the change rate on psychological health complaints differs among boys and girls, we included cross-level interaction terms between gender and both measures of family benefits (model 4). The results show that the absolute level of countries’ family benefits was significantly associated with girls’ likelihood of reporting 2 or more psychological health complaints. Figures 2a and b display these significant relationships for girls, indicating that the higher countries’ absolute level of family benefits (Fig. 2a), the lower the odds ratio of reporting 2 or more psychological health complaints among girls. In contrast to that finding, the higher countries’ change rate in family benefits (Fig. 2b), the higher the probabilities of health complaints among girls compared to boys.

With regard to social inequalities in health complaints, model 5 includes cross-level interaction terms of family benefits and family affluence. This interaction term are significantly related to complaints, highlighting that the impact of the absolute level of family benefits in 2009/2010 did significantly differ by family affluence.

The relative change rate in family benefits (2005/2006–2009/2010) varied by FAS, showing higher odds ratios for medium- and low affluent adolescents. Thus, social inequalities in health complaints were greater in countries with higher increases in family benefits since the beginning of the current economic recession. Due to the high correlation between national wealth (GDP per capita) and the relative change rate in public spending on family benefits, we also run the model without national wealth. The associations and coefficients remained the same. Figure 2 displays this association, showing that inequalities in psychological health complaints widened between family affluence groups.

## Discussion

This study examines for the first time the impact of public spending on family benefits on adolescent psychological health complaints and social inequalities in complaints during the recent economic recession in 27 European countries. The results show that the current absolute rate of public spending on family benefits in 2009/2010 was not related to adolescents’ psychological health complaints. In contrast, lower likelihoods of complaints were found for countries with higher relative change rates in family benefits (2005/2006–2009/2010). Contrary to our assumption, an increase in public spending on family benefits during the recession did not appear to reduce inequalities in young people’s health complaints. We further examined whether the impact of public spending on family benefits and the change rate in family benefits during the current recession on psychological health complaints differed between boys and girls. Our results showed that girls are more sensitive to the absolute level of family benefits and particularly to a higher increase in family benefits during the recession (2005/2006–2009/2010) compared to their male counterparts. In sum, our findings suggested that policy initiatives in terms of an increase in family benefits might partially benefit adolescent health, but tend to widen social inequalities in adolescent health during the recent recession. Although associations between increases in family benefits and psychological health complaints were small, our results are in line with other studies taking into account indicators at the country-/macro-level [24]. Studies using more distal characteristics, indicating that they are measured at higher levels, do have on average a smaller impact on young people’s health compared to more proximal determinants, such as individual background characteristics of children and adolescents.

Comparing and interpreting our findings with prior results is challenging as previous studies mainly focused on adult health, single-countries or individual-level associations, rather than on country-level outcomes of the recession as determinants of adolescent health and health inequalities among countries. For instance, Gili et al. [7] showed that frequencies of mood, anxiety, somatoform and alcohol-related disorders increased among adults in Spain during the current economic downturn (2006–2011) that was mainly caused by experiences with household unemployment and difficulties in mortgage payment. Another study from Greece also revealed a higher probability of reporting poor self-rated health during the recession (2006–2011) [8]. With regard to young people, a study using the Catalan Health Survey highlighted that health-related behaviours (e.g. eating habits, fast food consumption) and health-related quality of life improved among children and adolescents younger than 15 years. Further, social inequalities in young people’s obesity, mental health and health-related quality of life increased with decreasing parental education [17].

According to our results, the absolute level of public spending on family benefits in 2009 was not related to the magnitude of psychological health complaints, rather than the relative change rates in family benefits (2005/2006–2009/2010). This highlights the importance of (at least temporary) increases in family benefits in order to improve subjective health of better-off students. In this context, family benefits may have a limited effect on all families and children if paid on the basis of social security contributions, or on the conditions of work, thereby having a more limited coverage than universal benefits or services [25]. In general, the effects of benefits for families and young people vary depending on whether the money is spent on young people’s needs and whether the cash benefits target those children and adolescents who are the most disadvantaged. The most direct family policies are services and benefits-in kind delivered directly to young people, such as free childcare and preschool services, after school care or free school meals, whereas other countries have extended their services or reduced their fees [25].

With regard to social inequalities in psychological health complaints, we could not confirm our hypothesis of a buffering impact of public spending on family benefits on the extent of social inequalities in psychological health complaints among young people. Our results showed that inequalities in health complaints slightly widened among medium- and low-affluent adolescents living in countries with increasing levels of family benefits during the recent recession. A recently published Unicef Report on “The impact of the economic crisis on child well-being in rich countries” [26] highlighted that many European and OECD countries have increased their levels of family benefits during the recession, while others reduced their benefits for families [25–27]. For instance, Greece made a disparate system of child-related allowances into a less restrictive, more generous single benefit in 2013, while Latvia eased conditions for child-care benefits in 2014 and Poland increased amounts of family benefits and income ceilings in 2012–2013 [25, 26]. However, in several European countries, austerity policies were introduced. For instance, in Spain unemployment benefits and child-care benefits have been reduced, expenditures on social protection, particularly for families and children, declined from 5 to 3.5% between 2008 and 2011 [26]. The United Kingdom has implemented a series of cuts that reduced the real value and coverage of child benefits and tax credits for families with children since 2010 [25–27].

With regard to our study, our findings seem to be in line with results which have been published in a UNICEF Report on “Child Poverty and material deprivation in European Countries during the Great Recession” [28]. This report revealed that both the child poverty and the severe deprivation among young people were significantly lower in countries with more generous safety nets. Although social spending was associated with lower risks of child poverty at the start of the crisis (2008–2009), when many European countries implemented fiscal stimulus packages, social spending was no longer significantly related to child poverty and deprivation in later years of the recession (2010–2012), when many countries implemented austerity reforms. In particular, family benefits did not shield young people from risk of poverty and deprivation during the recession (2008–2012) [28].

Therefore, in line with these findings, we can conclude that social protection, particularly in terms of family benefits, is positively linked to a better overall psychological health among young people. However, a further increase in family benefits during the current economic recession seemed not to protect low affluent children and adolescents from suffering of psychological health complaints. This may be because protection through family benefits is not sufficient for low affluent families at the time when families struggle with circumstances that stem from the recession, such as loss of income or (fear of) unemployment. A further explanation could be that the majority of stimulating cash interventions during the current economic recession may have been temporary and may not target at those people in real need of social benefits. Thus, it is likely that public spending on family benefits fail the mission of tackling social inequalities within societies. In contrast, services or in-kind-benefits, such as free preschool, free school meals and after school care, are more likely to continue after times of economic recessions [25] and to mitigate social inequalities among young people. However, previous studies have shown that in countries with good formal social protection systems and high levels of social benefits, social inequalities in health did not widen during an economic recession [4, 10]. Another explanation is related to the overall social climate in Europe’s societies during the current economic recession. The societal, economic and labor market changes that resulted from the recession may also have consequences for the young people, their wellbeing and future prospects. Children and adolescents – particularly who are or feel themselves at the bottom end of the social ladder – are likely to feel anxious and psychologically stressed when parents or family members face unemployment or income loss during this insecure period and when they suffer downturns [26, 29], resulting in higher prevalence of psychological health complaints among low affluent young people.

The HBSC study presents an outstanding opportunity to analyse cross-national patterns of health and health inequalities among young people. The strengths of this study include the use of a large cross-national dataset and standardized data collection. However, there were methodological issues that may have influenced our results and should therefore be considered. One possible limitation is the application of self-reported measures of health which may vary by country, culture and socio-economic position. However, another study could show that self-reported health measures did not differ among countries in terms of socio-economic position [19]. It should also be acknowledged that the Family Affluence Scale measures only one dimension of social position. Previous studies have shown that family affluence can be applied as a proxy for individual social position [18, 30]. Further, a previous study showed that the four FAS items completed by 11-years olds corresponded with parents’ responses and were correlated with parents’ occupational status [31]. Additionally, FAS was closely related to national income at the aggregated level [32]. As we used only one indicator of social spending, we conducted sensitivity analyses by using another indicator of social spending (health expenditures, measured as a percentage of GDP). As for both measures of public spending on family benefits (absolute level in 2009/2010 and relative change rate in family benefits between 2005/2006–2009/2010), a similar pattern was obtained for the impact of the absolute level of health expenditure as well as for the change rate in health expenditure on the likelihoods of reporting 2 or more psychological health complaints (see Web Additional file 2: Table S1 and Additional file 3: Figure S1). According to our findings for all social spending indicators, there seems to be a systematic structure of welfare spending. Another limitation was the short duration of the study by analyzing the impact of levels of public spending on family benefits in 2009/2010 and the relative increase, decrease or stagnation in family benefits (2005/2006–2009/2010) on adolescent health and health inequalities. In this context, the effects of the current economic recession on health and particularly on health inequalities may take several years to observe [33].

## Conclusions

Our findings highlight the importance of social security policies for young people’s magnitude of psychological health complaints, whereas a buffering effect by the extent of social spending on family benefits on inequalities in adolescent health complaints could not be revealed rather than widening inequalities in health in countries with higher increases in family benefits during the recent economic recession. Thus, this study suggests that governments might be able to protect their young populations and future generations, especially by budgeting and public spending on social security for better-off youth. In contrast, health inequalities were not ameliorated by those initiatives. Particularly in times of economic recession, these results provide a specific opportunity for stimulus packages for policy makers and health promotion initiatives in order to invest not only in cash-benefits for social security, but also in in-kind-benefits, such as family and parenting support programs [25, 34]. The recognition of the health and social consequences of economic austerity packages must be a priority in further shaping economic and fiscal policies in European countries [35].

## Additional files

Additional file 1: Information on ethical approvals in the HBSC study 2009/2010). (DOCX 19 kb) Additional file 2:
**Table S1.** Associations between public expenditures on health (2009/2010), relative change in public expenditures on health (2005/2006–2009/2010) and psychological health complaints, HBSC 2009/2010). (DOCX 19 kb) Additional file 3:
**Figure S1.** Predicted probabilities of two or more (at least weekly) psychological complaints (*N* = 144,754), according to the relative percentage change in health expenditures (2005/2006–2009/2010) across 27 countries, stratified by family affluence, HBSC 2009/2010). (DOCX 14 kb)

## Abbreviations

FAS: Family affluence scale
GDP: Gross domestic product
HBSC: Health behavior in school-aged children
ICC: Intraclass-correlation coefficient
OECD: Organisation for economic co-operation and development
Pc: per capita
PPP: in constant prices and constant purchasing power parity
SOCX: Social expenditure database
