# Supplementary material for: Adolescents’ psychological health during the economic recession: does public spending buffer health inequalities among young people?
Source: BMC Public Health. 2016 Aug 24;16(1):860. doi: 10.1186/s12889-016-3551-6 (PMC4995668; doi:10.1186/s12889-016-3551-6)
Supplement: Additional file 1: — Information on ethical approvals in the HBSC study 2009/2010). (DOCX 19 kb) [file 12889_2016_3551_MOESM1_ESM.docx]

### Additional file 1:

### Information on ethical approvals in the HBSC study 2009/2010 ^1^

| **Country (n=27)** | **Survey year 2009/2010** |  |
| --- | --- | --- |
| **Austria** | na | - |
| **Belgium (Flanders)** | yes | Ethics review committee of the University Hospital of Ghent |
| **Belgium (Wallonia)** | yes | Education Boards of the different School Networks in the Federation Wallonia-Brussels |
| **Czech Republic** | not required | - |
| **Denmark** | not required | - |
| **England (UK)** | yes | Ethics Committee of University of Hertfordshire |
| **Estonia** | yes | Tallinn Medical Research Ethics Committee, Tallinn, Estonia |
| **Finland** | yes | Trade Union on Education in Finland and the National Board of Education |
| **France** | na | - |
| **Germany** | yes | Ethics Committee of the Medical Association of Hamburg (on behalf of the University Clinic Hamburg) |
| **Greece** | yes | Ministry of Education  Pedagogical Institute |
| **Hungary** | yes | Scientific and Research Ethical Committee of the Medical Research Council of the Hungarian Ministry of Health |
| **Ireland** | yes | Human Research Ethics Committee of the National University of Ireland, Galway |
| **Iceland** | not required | - |
| **Italy** | yes | Ethical Board of the Istituto Superiore di Sanità, Roma |
| **Latvia** | not required | - |
| **Lithuania** | yes | Kaunas Regional Biomedical Research Ethics Committee |
| **Luxemburg** | yes | Comité national d Ethique de Recherche (CNER), Comite National pour la Protection des Données (CNPD) |
| **Netherlands** | na | - |
| **Norway** | na | - |
| **Poland** | yes | Ethics Committee at the National Research Institute of Mother and Child, Warsaw |
| **Portugal** | yes | Hospital S Joao, O Porto University, Ethical committee |
| **Romania** | na | - |
| **Scotland (UK)** | yes | University of Edinburgh, School of Education, Ethics Committee |
| **Slovakia** | yes | Ethics Committee of the Medical Faculty at the P. J. Safarik University in Kosice |
| **Slovenia** | yes | Republic of Slovenia National Medical Ethics Committee |
| **Spain** | na | - |
| **Sweden** | not required | - |
| **Switzerland** | yes | Commission cantonale (VD) d’éthique de la recherche sur l’être humain |
| **Wales (UK)** | yes | Ethics Committee of Cardiff University, School of Social Sciences |

^1^ Information provided by the principal investigator of each country. The list of principal investigators at the time of the survey can be found in the acknowledgments; na = not available
