# Supplementary material for: Adolescents’ psychological health during the economic recession: does public spending buffer health inequalities among young people?
Source: BMC Public Health. 2016 Aug 24;16(1):860. doi: 10.1186/s12889-016-3551-6 (PMC4995668; doi:10.1186/s12889-016-3551-6)
Supplement: Additional file 2: — Table S1. Associations between public expenditures on health (2009/2010), relative change in public expenditures on health (2005/2006–2009/2010) and psychological health complaints, HBSC 2009/2010). (DOCX 19 kb) [file 12889_2016_3551_MOESM2_ESM.docx]

**Additional file 2:**

**Table S1: Associations between public expenditures on health^#^ (2009/2010), relative change in public expenditures on health^#^ (2005/2006-2009/2010) and psychological health complaints (HBSC 2009/2010)**

|  | **Empty model (M1)** | **Individual variables (M2)** | **Macro-level variables (M3)** | **Model with cross-level interactions (M4)** |
| --- | --- | --- | --- | --- |
|  | OR (95% CI) | OR (95% CI) | OR (95% CI) | OR (95% CI) |
| **Individual variables** |  |  |  |  |
| Sex (Ref.: *boys*) |  | 1 | 1 | 1 |
| Girls |  | 1.708*** | 1.708*** | 1.708*** |
|  |  | (1.66-1.75) | (1.66-1.75) | (1.66-1.75) |
|  |  |  |  |  |
| Age (Ref.: *11 years*) |  | 1.000 | 1.000 | 1.000 |
| 13 years |  | 1.289*** | 1.289*** | 1.289*** |
|  |  | (1.25-1.33) | (1.25-1.33) | (1.25-1.33) |
| 15 years |  | 1.486*** | 1.486*** | 1.485*** |
|  |  | (1.44-1.54) | (1.44-1.54) | (1.44-1.53) |
| Family affluence (Ref.: *high*) |  | 1.000 | 1.000 | 1.000 |
| Medium |  | 1.129*** | 1.129*** | 1.130*** |
|  |  | (1.10-1.17) | (1.10-1.17) | (1.10-1.17) |
| Low |  | 1.386*** | 1.386*** | 1.385*** |
|  |  | (1.34-1.43) | (1.34-1.43) | (1.34-1.43) |
| **Macro-level variables** |  |  |  |  |
| National wealth in 2009/2010 (GDPpc) |  |  | 1.000  (0.99-1.00) | 1.000  (0.99-1.00) |
|  |  |  |  |  |
| Youth unemployment rate (2009/2010) |  |  | **1.018*****  **(1.01-1.04)** | **1.018*****  **(1.01-1.04)** |
|  |  |  |  |  |
| Health expenditure in 2009/2010 |  |  | **0.917+** | **0.919+** |
|  |  |  | **(0.83-1.00)** | **(0.84-1.00)** |
|  |  |  |  |  |
| relative change in health expenditure**^#^** (2005/2006-2009/2010) |  |  | **0.999+** | 0.998 |
|  |  |  | **(0.98-1.00)** | (0.99-1.01) |
| **Cross-level interactions** |  |  |  |  |
| Health expenditure in 2009/2010  x high FAS (Ref.) |  |  |  | 1.000 |
| x medium FAS |  |  |  | 1.003 |
|  |  |  |  | (0.98-1.03) |
| x low FAS |  |  |  | 0.985 |
|  |  |  |  | (0.96-1.01) |
| relative change in health expenditure**^#^** (2005/2006-2009/2010)  x high FAS (Ref.) |  |  |  | 1.000 |
| x medium FAS |  |  |  | 1.002 |
|  |  |  |  | (0.99-1.01) |
| x low FAS |  |  |  | **1.003*** |
|  |  |  |  | **(1.00-1.01)** |
|  |  |  |  |  |
| Constant | 0.261***  (0.23-0.30) | 0.136***  (0.12-0.16) | 0.135***  (0.12-0.15) | 0.134***  (0.12-0.15) |
| ICC (country-level) | 0.0375=3.75% | 0.0386=3.85% | 0.0234=2.34% | 0.0233=2.33% |
| N (Individuals) | 144,754 | 144,754 | 144,754 | 144,754 |
| N (Countries) | 27 | 27 | 27 | 27 |

Note: + p<0.10, * p<0.05, ** p<0.01, *** p< 0.001. Country-level indicators are centered on the Grand-Mean.

**^#^** Public expenditure on health was measured as percentage of GDP (Source: World Bank Databank, http://data.worldbank.org/indicator/SH.XPD.PUBL.ZS/countries?page=2&display=default)
