# Supplementary material for: Adolescents’ psychological health during the economic recession: does public spending buffer health inequalities among young people?
Source: BMC Public Health. 2016 Aug 24;16(1):860. doi: 10.1186/s12889-016-3551-6 (PMC4995668; doi:10.1186/s12889-016-3551-6)
Supplement: Additional file 3: — Figure S1. Predicted probabilities of two or more (at least weekly) psychological complaints (N = 144,754), according to the relative percentage change in health expenditures (2005/2006–2009/2010) across 27 countries, stratified by family affluence, HBSC 2009/2010). (DOCX 14 kb) [file 12889_2016_3551_MOESM3_ESM.docx]

**Additional file 3:**

**Figure S1: Predicted probabilities of two or more (at least weekly) psychological complaints (N=144,754), according to the relative percentage change in health expenditures* (2005/2006-2009/2010) across 27 countries, stratified by family affluence (HBSC 2009/2010)**

Note: * Public expenditures on health was measured as percentage of GDP (Source: World Bank Databank, <http://data.worldbank.org/indicator/SH.XPD.PUBL.ZS/countries?page=2&display=default>)
